# fNIRS measurement of cortical activation and functional connectivity during a visuospatial working memory task

**DOI:** 10.1371/journal.pone.0201486

**Published:** 2018-08-02

**Authors:** Joseph M. Baker, Jennifer L. Bruno, Andrew Gundran, S. M. Hadi Hosseini, Allan L. Reiss

**Affiliations:** 1 Center for Interdisciplinary Brain Sciences Research, Division of Brain Sciences, Department of Psychiatry and Behavioral Sciences, Stanford University School of Medicine, Stanford, California, United States of America; 2 Department of Radiology, Stanford University School of Medicine, Stanford, California, United States of America; Taipei Veterans General Hospital, TAIWAN

## Abstract

Demands on visuospatial working memory are a ubiquitous part of everyday life. As such, significant efforts have been made to understand how the brain responds to these demands in real-world environments. Multiple brain imaging studies have highlighted a fronto-parietal cortical network that underlies visuospatial working memory, is modulated by cognitive load, and that appears to respond uniquely to encoding versus retrieval components. Furthermore, multiple studies have identified functional connectivity in regions of the fronto-parietal network during working memory tasks. Together, these findings have helped outline important aspects of the neural architecture that underlies visuospatial working memory. Here, we provide results from the first fNIRS-based investigation of fronto-parietal signatures of cortical activation and functional connectivity during a computer-based visuospatial working memory task. Our results indicate that the local maxima of cortical activation and functional coherence do not necessarily overlap spatially, and that cortical activation is significantly more susceptible to task-specific demands compared to functional connectivity. These results highlight important and novel information regarding neurotypical signatures of cortical activation and functional connectivity during visuospatial working memory. Our findings also demonstrate the utility of fNIRS for interrogating these cognitive processes.

## Introduction

The everyday world is wrought with demands on our visuospatial working memory. For instance, as we drive to work we encode and maintain the spatial position of other drivers on the road–as well as our coffee in the center console–in our working memory. Accurate visuospatial representation of those objects in working memory allows us to navigate our car through traffic, while reaching for our coffee without incident. This is possible because of highly evolved and specialized cognitive abilities that allow us to successfully negotiate these demands, most of the time. Brain science researchers interested in the biological underpinnings of these processes have worked to elucidate the neural foundations of visuospatial working memory in humans. These efforts have helped identify the patterns of cortical activation and functional coherence within the fronto-parietal network that occur in concert and ultimately facilitate visuospatial working memory.

Converging evidence from multiple studies using functional magnetic resonance imaging (fMRI) and functional near-infrared spectroscopy (fNIRS) have identified specific regions of the prefrontal and parietal cortices that are involved in visuospatial working memory. In particular, fMRI-based studies have identified significant activation in the bilateral dorsolateral prefrontal cortex and the bilateral superior parietal cortex during visuospatial working memory tasks [1]. Convergent evidence of cortical activation within these regions has also been identified using fNIRS; Tsujimoto and colleagues required participants to memorize the spatial position of two or four sample cues presented in a concentric circle in the center of a computer screen. As hypothesized, this activity elicited significant increases in oxygenated hemoglobin within the bilateral prefrontal cortex [2]. Moreover, using a line orientation task, Herrmann and colleagues identified significant bilateral activation of the inferior and superior parietal cortex using fNIRS [3]. Furthermore, multiple studies have reported differential patterns of cortical activation in the fronto-parietal network during encoding compared to retrieval of information in memory [4–7].

While analysis of cortical activity provides useful information, a complete understanding of the neural correlates to visuospatial working memory cannot be ascertained without the joint analysis of cortical activation and functional connectivity. Indeed, the interrogation of frontal-parietal connectivity has uncovered important signatures of structural and functional connectivity that directly impact visuospatial working memory. For example, using single-cell recordings in monkeys, Salazar and colleagues demonstrated widespread, task-dependent, and content-specific synchronization of activity across the fronto-parietal network during visual working memory [8]. These results indicate that short-term memories are represented by large-scale patterns of synchronized activity across the frontal-parietal network. Moreover, working memory training has been shown to impact structural connectivity in the brain, indicating that a causal relationship exists between working memory ability and neural connectivity [9].

The combined analysis of cortical activation and functional connectivity provides a rich view of the neural architecture underlying visuospatial working memory that thoroughly details the roles that the fronto-parietal network plays during this ubiquitous cognitive process. However, to date no published report provides a concurrent evaluation of cortical activation and functional coherence during a visuospatial working memory task. Here, we address this shortcoming directly by providing the initial framework for the use of fNIRS to investigate concurrent signatures of cortical activation and functional connectivity during a computer-based visuospatial working memory task. Furthermore, in an effort to assess the cortical signatures of visuospatial processing at or near the limits of our participant’s perceptual abilities, we employed a novel approach to task difficulty calibration that is based on individual participants’ visuospatial psychophysical sensitivity. The details of this task are provided below.

## Methods

### Participants

A total of fifteen (n_female_ = 8, range = 18-36yrs) healthy adults were recruited for participation. All participants were right handed (Edinburg Handedness Inventory mean = 78.75, sd = 40.214) and had normal or corrected to normal vision and hearing. Participants were excluded if they reported any clinical psychiatric history such as anxiety or depression, or other chronic or significant medical conditions. Consumption of recreational stimulants (e.g., caffeine, tobacco, etc.) prior to participation was not controlled. Informed consent was obtained from all participants prior to participation. The protocol was approved by the Stanford University Institutional Review Board, and all clinical investigation was conducted in accordance with the principles expressed in the Declaration of Helsinki.

### Materials

All stimuli were generated using Matlab 2014b Psychtoolbox, and were presented on a MacBook Pro connected to a 20” LED monitor. Task responses were collected by button presses on a standard English language QWERTY keyboard. Hemodynamic activity was recorded using a 40-channel tandem NIRsport (NIRx, Germany) time-domain fNIRS device with a sampling rate of 7.8Hz. A total of 32 (n_sources_ = 16, n_detectors_ = 16) fNIRS optodes were distributed evenly over the left prefrontal, right prefrontal, left parietal, and right parietal cortices. The optodes were held securely to the participant’s head by way of individually sized caps designed for neuroimaging applications (Brain Products, Germany). The optode locations were cut into each cap at size-specific International 10/20 system locations [10,11]. Plastic supports placed between each source/detector pair that constituted a recording channel maintained a 3cm channel length for all participants. In this manner, the arrays were consistently placed on specific 10/20 locations of interest despite changes in head size across participants [11,12]. This consistency allowed us to subset the fNIRS channels of interest down to those directly measuring each region of interest (see 2.4.3).

### Procedure

Each participant was seated in front the computer screen and keyboard. Participants were positioned such that their eyes were 70cm from the screen, and the stimulus array subtended a maximum visual angle of 10.61°. Next, the participants completed a *just noticeable difference* estimation task described below (2.3.1). Following the estimation task, the participants were fitted with the fNIRS cap, after which each participant completed the visuospatial working memory task (2.3.2). On average, participation in our study lasted roughly 1.16 hours with approximately 15 minutes dedicated to the just noticeable difference estimation task, 5 minutes of rest, 20 minutes of fNIRS set-up, and 30 minutes to complete the fNIRS task.

### Just noticeable difference estimation task

In order to account for individual differences in visuospatial psychophysical sensitivity during the fNIRS task, the just noticeable difference (JND) needed for each participant to distinguish a change in the location of a dot was assessed prior to the fNIRS scan. An individual’s JND defines the amount of deviation required to identify a difference between two continuous quantities [13,14]. For our task, the continuous quantity of interest was the amount of spatial deviation required in the target-to-original location of a single dot in an array for each participant to reliably identify a change in spatial position. Thus, the JND estimation task acted to optimize the task difficulty for each participant. This is important, as psychophysical discrimination of continuous quantities at or near the JND correlates with increased neural activation [15–23]. Conversely, the use of an arbitrary task difficulty for all participants would likely result in varying perceived task difficulties, which in turn may unexpectedly influence cortical activations.

**Fig 1:**
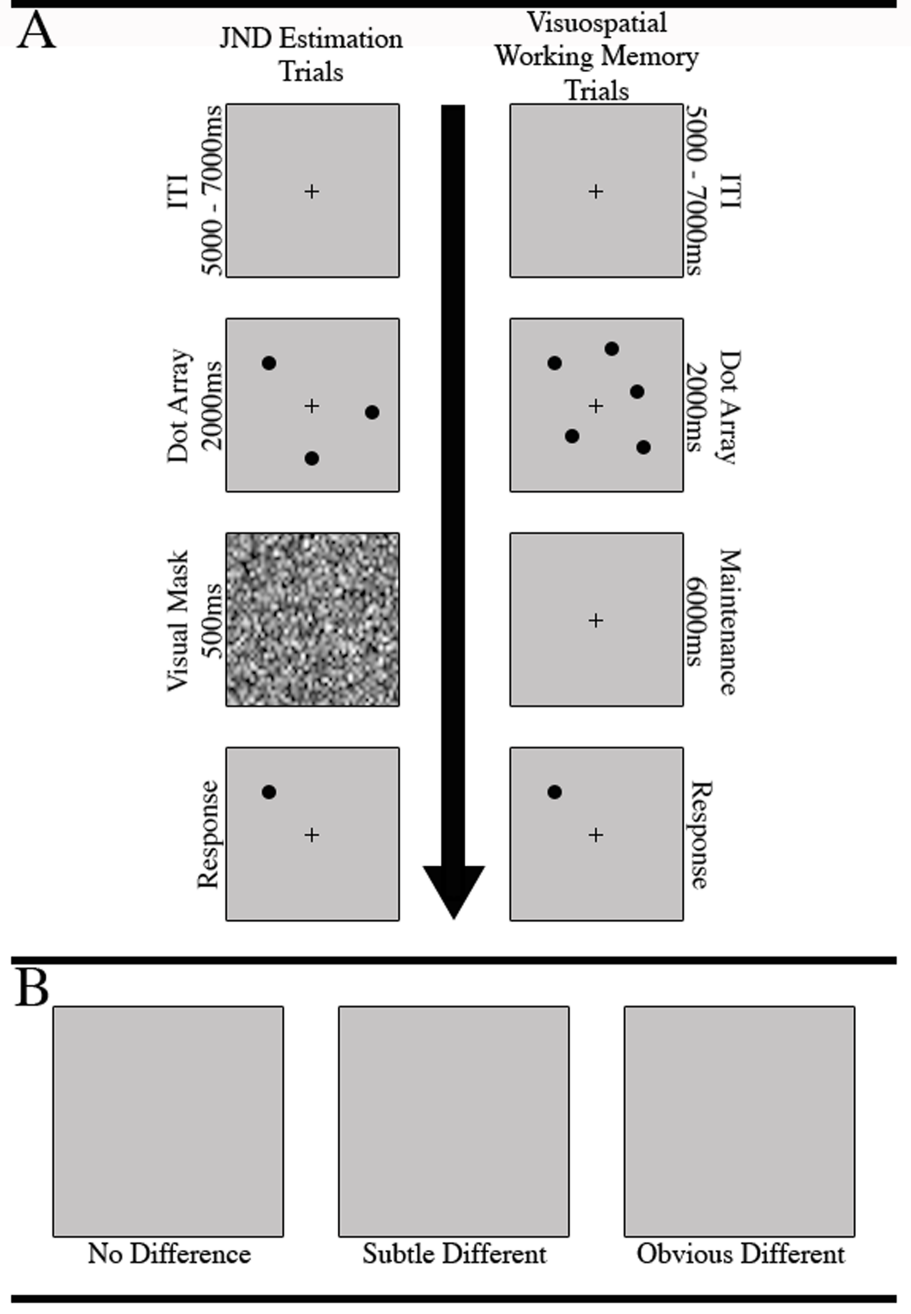
Trial structure for JND and fNIRS task trials. A. Participants completed 200 JND estimation trials. Each trial began with a 5–7 second ITI, and was followed by a 3 or 5 dot array. Next, a visual mask was displayed for .5s, and was immediately followed by the single-dot presentation, at which point the participant could respond. The fNIRS task trails proceeded in a similar fashion, except the visual mask portion was extended to 6s and the screen simply remained blank except for a central cross; B. fNIRS task difficulty was modulated by the spatial difference between the original target dot location within its array and its location on the response screen. Spatial deviations were defined as “no difference”, “subtle difference” (JND x 0.1), and “obvious difference” (JND x 0.8). The updated location of the target dot was randomly selected from a vector of x- and y-axis values that constitute a circle, with a radius equal to the spatial deviation in pixels, that lay around the center of the original dot.

The JND estimation task included 200 trials (Fig 1A): On each trial, an array of three or five non-overlapping dots was presented for 2 seconds in a random concentric pattern around a fixation cross in the center of a computer screen. Next, a visual mask was briefly presented for 500ms. The luminance of the visual mask was matched to that of the dot array and acted to discourage participants from responding based on low-level perceptual strategies such as maintaining a negative after-image of the dot array following its 2-second display. Thus, in order to accurately identify changes in the target dot location, participants were required to rely on their working memory of the dot locations and engage their visuospatial perceptual discrimination abilities to determine if the location had changed.

Immediately following the visual mask, a single (i.e., target) dot was presented on the screen in either the same location (n_trials_ = 20) as one of the previous dots, or in a different location (n_trials_ = 160). A third classification of trials (n_trials_ = 20) presented an obvious spatial deviation of 200 pixels (4.33°) (Fig 1B). The participants were required to determine if the target dot was presented in the same (press ‘z’) or different (press ‘x’) location than its presentation within the original array. In order to quickly estimate each participant’s JND, we employed a stair step procedure to dynamically modulate the difficulty of each trial [24]. That is, on trials in which a change in target dot location occurred, the distance of the target dots deviance from its original position was determined by the previous trial’s performance.

**Fig 2:**
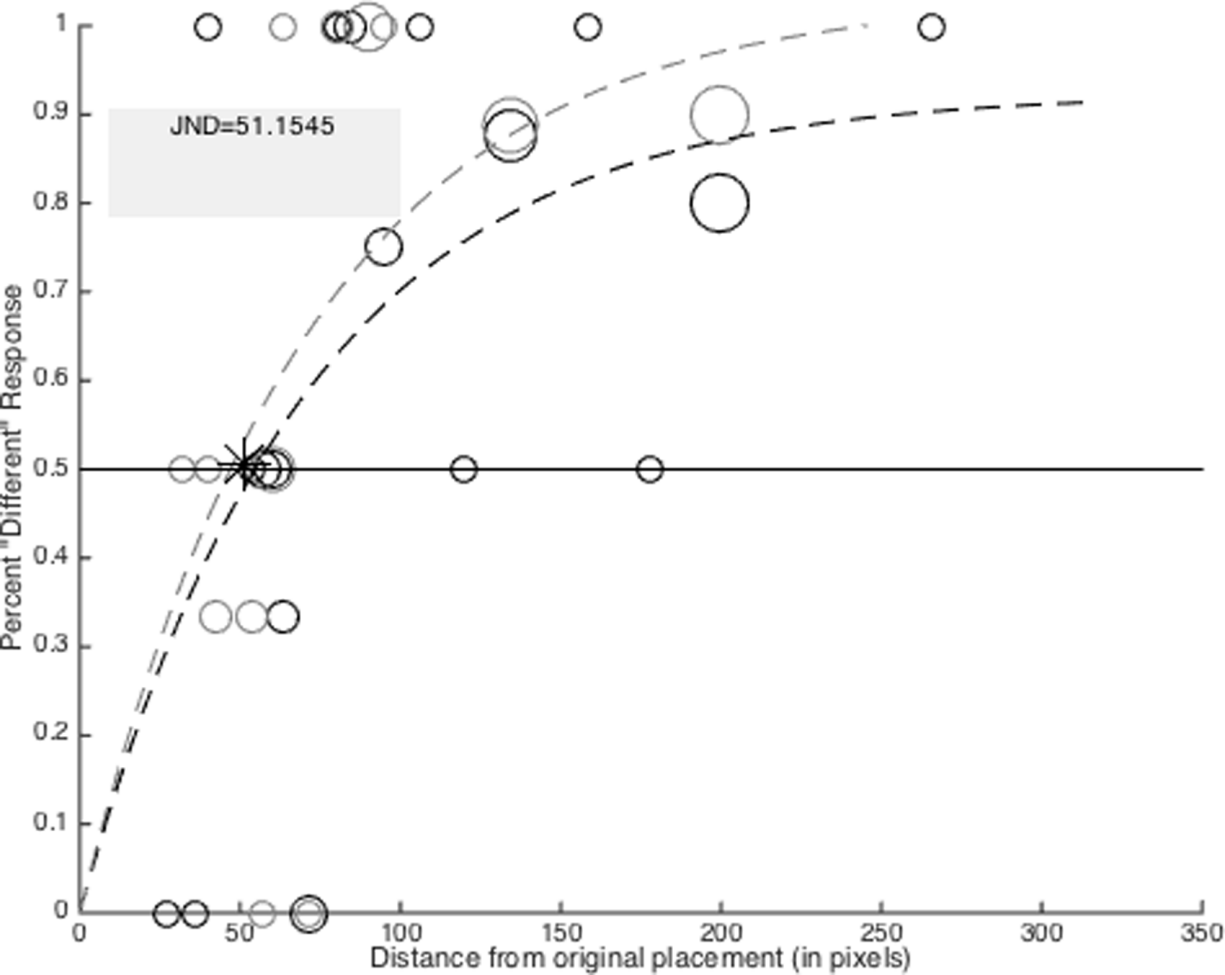
JND output example. Outcome of JND estimation task for a single participant. The light and dark circles and dotted lines represent JND trials for 3 and 5 dot conditions respectively. The size of each dot represents the number of trials experienced at that distance from the original position of the target dot location. Each staircase procedure began with a spatial deviation of 150 pixels. The JND was calculated as the average point along the x-axis at which both non-linear trend lines crossed the 50% correct response point along the y-axis. The JND is marked with a ‘*’.

All participants began with an initial target-to-original deviation of 150 pixels (3.24°). Correct responses (i.e., correct identification of a change in the target-to-original position) reduced the spatial deviations on the successive trial by 33%, thus making the next trial more difficult. Conversely, incorrect responses increased the spatial deviation by 33%, making the next trial easier. Responses to same location or obvious spatial deviation trials did not have any bearing on subsequent trial difficulty. That is, regardless of the participant’s performance on these trial types, the target-to-original position was not modified. The updated location of the target dot was randomly selected from a vector of x- and y-axis values that constitute a circle, with a radius equal to the spatial deviation in pixels, that lay around the center of the original target dot. Thus, the target dot had an equal probability of reappearing in any direction around its original placement. The order of trial types within the JND task was randomized prior to data collection, and the same randomized order was used across each participant (i.e., pseudorandomization). In order to collect multiple samples of trial performance across the entire range of possible deviations, the stair step procedure was reset every 10 trials in which a spatial change occurred. Next, a non-linear weighted regression model was used to fit a non-linear trend line to participant’s response accuracy at each deviation distance. The JND was calculated as the point on the trend line corresponding to 50% accuracy (Fig 2).

### Visuospatial working memory task

The fNIRS visuospatial working memory task proceeded much like the JND estimation task (Fig 1A). A total of 124 trials began with the presentation of a 3 or 5 dot array presented in a random concentric circle around a central fixation cross [2,25]. The dot array remained visible for 2 seconds, after which it disappeared for 6 seconds. Next, a single target dot was presented in either the same (n_NoChange_ = 40) or different location as one of the dots in the previous array. The amount of target-to-original change was defined as *subtle different* (n_SubtleDifferent_ = 28), or *obvious different* (n_ObviousDifferent_ = 28) based on the participants visuospatial JND. Subtle different changes were defined as a target-to-original deviation of $JND+\;\left(JND\;x\;0.1\right)$, whereas obvious different trials were calculated as $JND+\;\left(JND\;x\;0.8\right)$. Participants were instructed to press the ‘z’ button if they did not detect a change in target-to-original dot location, or to press the ‘x’ button if a change was detected. Furthermore, 20 “control” trials were presented, which began with the presentation of 3 or 5 squares or triangles. On control trials (n_Control_ = 28), participants were instructed to disregard the spatial position of the objects, and instead press the ‘z’ button if the shape appearing in the retrieval portion of the trial was a square, or the ‘x’ button if the shape was a triangle. Control trial responses were not dependent on the shape or position of the objects in the original array. Thus, control trials provided identical visual information and required the same physical response, but did not engage visuospatial working memory processes. The order of each trial type was pseudorandomized.

## Statistical analyses

### Analysis of behavioral results

Repeated measures analysis of variance (ANOVA) and FDR corrected pairwise comparisons were used to assess the influence of dot number and trial difficulty on task accuracy and response time.

### General linear model analysis of cortical activation

The differential patterns of cortical activation that occurred throughout our task were assessed using a general linear model (GLM) approach. The use of GLM for analysis of event-related fNIRS designs has been well established [26,27]. All preprocessing and analysis of fNIRS data was conducted using the HomER2 package in Matlab [28]. First, all optical density data were corrected for motion artifacts by the use of a wavelet motion correction procedure [29]. Next, the optical density data were bandpass filtered between 0.01 and 0.5 Hz prior to being converted to oxygenated hemoglobin (HbO) and deoxygenated hemoglobin (HbR) values using the modified Beers-Lambert law [30]. The HbO values resulting from this conversion were used for each analysis reported below. Identical analyses using HbR data are reported as Supporting Information.

The onset and duration of the task specific elements related to each analysis were submitted to the GLM procedure as predictor variables. The GLM procedure assumed a canonical hemodynamic response function and Gaussian error structure. The onset and durations variables were used to estimate standardized beta coefficients for each condition within the control and non-control trials respectively. The sign and magnitude of each beta coefficient provides an indicator of the direction (positive/negative) and intensity of change in hemoglobin oxygenation (i.e., cortical activity) that occurred during each condition. In order to capture the cortical activation that was unique to the task demands, and thus not expected to be present in signals corresponding to the control conditions, contrasts were made between the coefficients estimated for each condition and their respective control. The outcome of these contrasts were then submitted to the functional localization procedure described below.

### Functional localization within each region of interest

**Fig 3:**
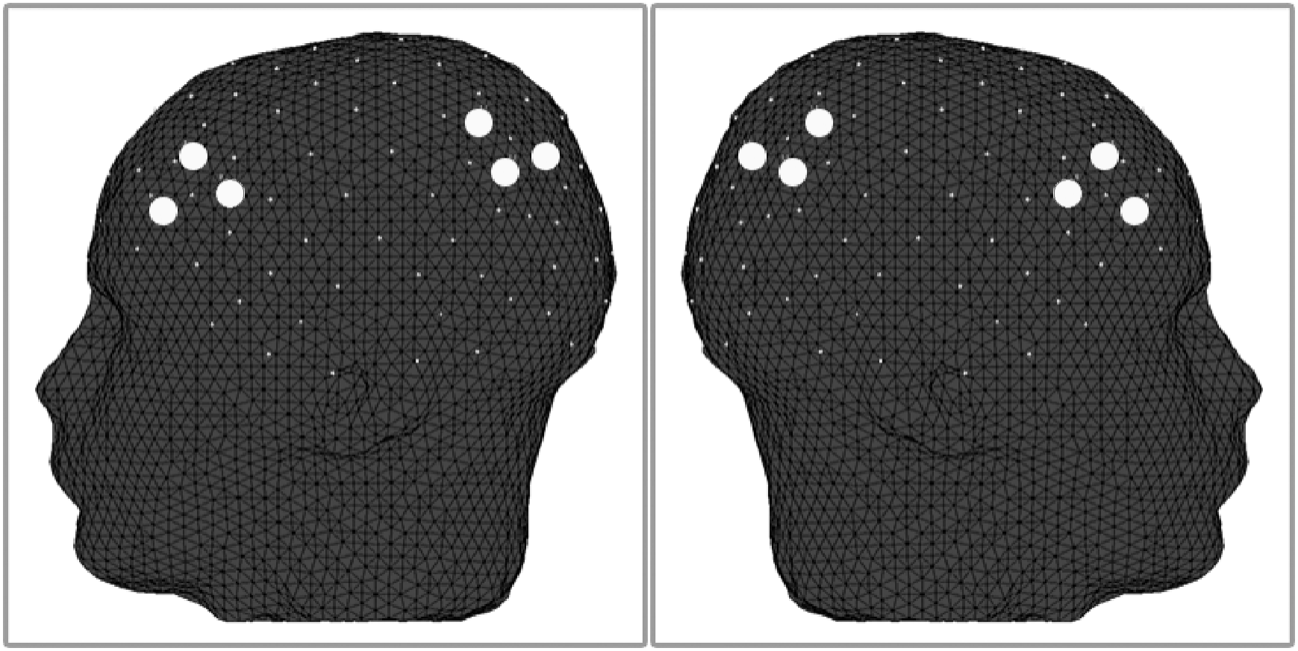
Location of regions of interest. A total of three channels were isolated within each fNIRS optode patch to target the bilateral dorsolateral prefrontal cortices and bilateral intraparietal sulci. The approximate position of each channel (large dots) is shown here. The International 10–20 system was used to identify the position of each channel.

Based on the literature described above, we selected four a priori regions of interest. These regions included the left dorsolateral prefrontal cortex (lPFC), the right dorsolateral prefrontal cortex (rPFC), the left intraparietal sulcus (lPAR), and the right intraparietal sulcus (rPAR). A total of three fNIRS channels recorded hemodynamic activity within each region of interest, resulting in 12 total channels of interest (Fig 3). In order to reduce the overall spatial coverage included in our analysis of cortical activation, we employed a functional localization procedure within each participant that identified the single channel within each region of interest that responded the greatest to each condition. Functional localization was accomplished by selecting the largest mean beta contrast for each condition of interest across all three channels within each region of interest. The localized channel within each region of interest was then submitted for group-level statistical analysis.

### Wavelet transform coherence analysis

Wavelet transform coherence (WTC) is a method of analyzing the coherence and phase lag between two time series as a function of both time and frequency. WTC is based on the continuous wavelet transform, which decomposes a single time series in time-frequency space by successively convolving the time series with scaled and translated versions of a wavelet function [31,32]. WTC is well suited to investigate non-stationary changes in coupling between fNIRS time-series, and has been used to identify both intra- and inter-brain dynamics across multiple tasks [33,34]. WTC was calculated using the Wavelet Coherence Toolbox for MATLAB [35].

Here, WTC was calculated for all channel pairings *within* (e.g., between all channels within the left prefrontal region of interest) and *between* each region of interest (e.g., between all channels within the left prefrontal and all other regions of interest), resulting in 144 inter-channel WTC decompositions per participant. The optical density data entered into each WTC decomposition were unfiltered and were not motion corrected. In order to normalize the statistical distribution of the values within each coherence decomposition array, each value was subject to Fischer z-transformation [33,34]. Next, for each condition of interest, the data within each decomposition array were reduced to the condition-relevant frequency band and time. Based on our task structure, the frequency band of interest was between 7s and 19s, corresponding to frequency 0.143Hz and 0.053Hz respectively. The mean of each condition-relevant data subset was then calculated and submitted for statistical analysis.

## Results

### Behavioral results

**Fig 4:**
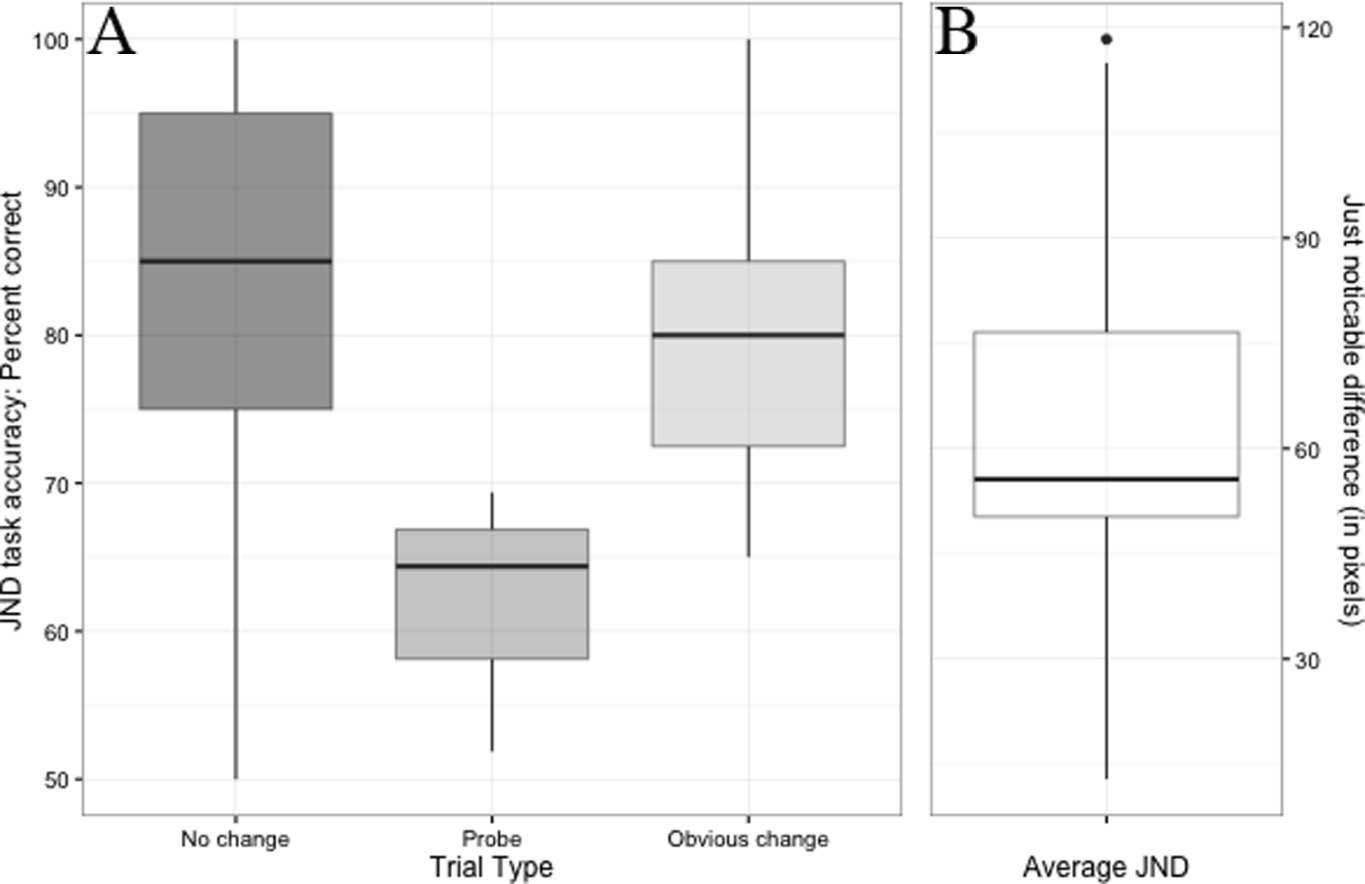
JND estimation task performance. A. Inter-quartile range of participant’s JND estimation task performance for each trials type. The top and bottom of each box represent the 75^th^ and 25^th^ percentile of performance respectively. The bold line within each box represents the median (i.e., 50^th^ percentile) task performance. The length of each whisker represents the extent of the lowest and highest datum that lie within +/- 1.5 times the inter-quartile range. Data points outside of this range are labeled with dots. Performance was significantly lower on probe trails compared to both “no change” and “obvious change” trials. B. Inter-quartile range of participant’s JND. The median JND for our task was 56.41 pixels.

Participant’s performance on the JND estimation task shows a clear and significant drop in task accuracy for probe trials compared to both “No change” (*p* = .0008, Cohen’s *d* = 1.8) and “Obvious change” (*p* < .0001, Cohen’s *d* = 2.19) trials (Fig 4A). This drop in performance is expected as probe trials reached or exceeded the participant’s JND. The median JND on our task was 56.41 pixels (Fig 4B).

**Fig 5:**
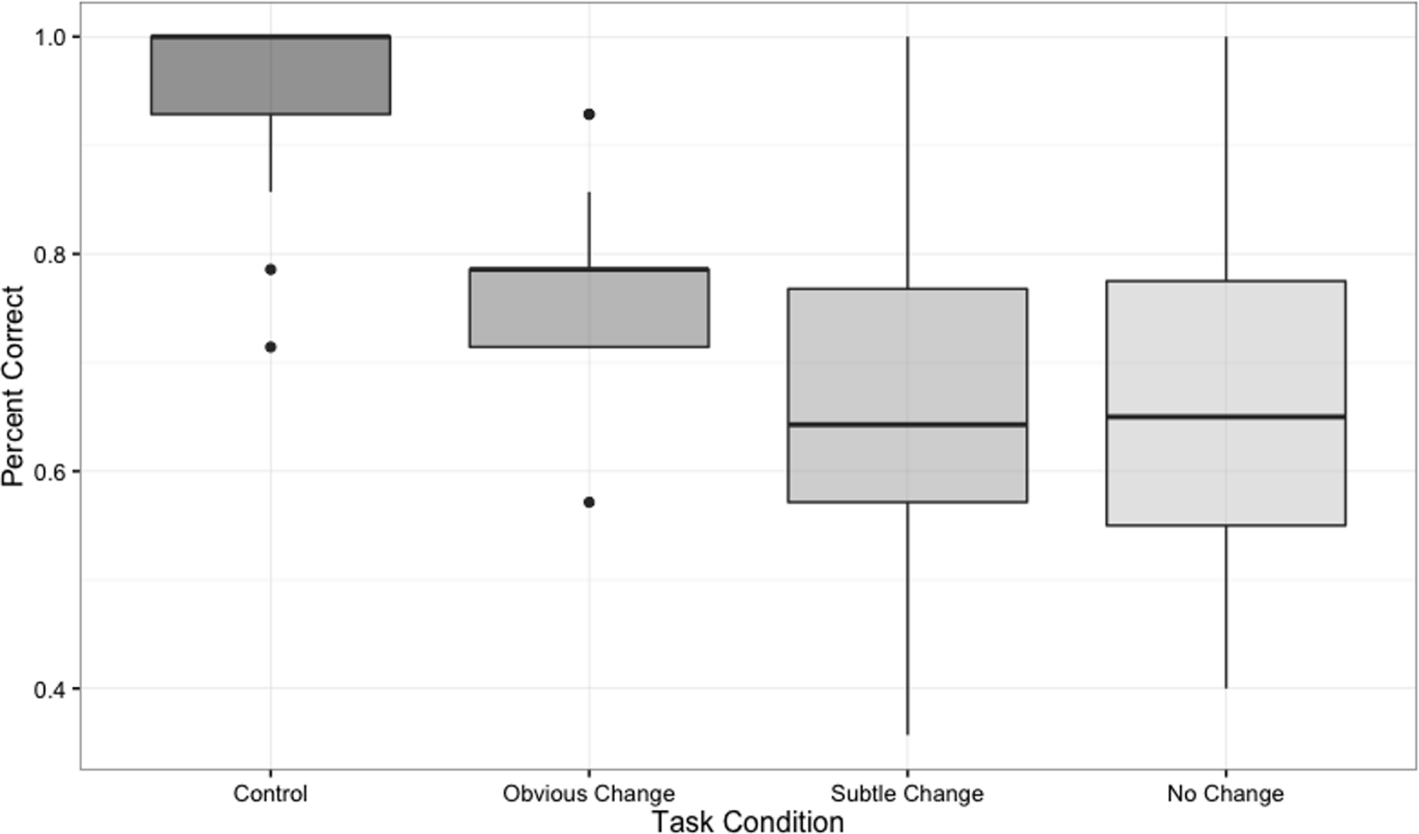
fNIRS task behavioral performance. Inter-quartile ranges of fNIRS task performance for each trial type. Task performance followed the predicted pattern of highest median performance on control trials, followed by “obvious change”, “subtle change”, and “no change” trial types in that order.

Differences in task accuracy were assessed across the three vs. five dot trials, as well as between subtle different, obvious different, no change, and control trials. A 2 (dot number) x 4 (trial type) repeated measures ANOVA identified a significant main effect of trial type on task accuracy (*F*(3, 104) = 18.419, *MSE* = 0.451, *p* < .001, *partial η*^*2*^ = 0.27). Follow-up pairwise t-tests with FDR correction identified significant performance differences between control trials and obvious different trials (FDR *p* = .005, Cohen’s *d* = 0.53), control trials and subtle different trials (FDR *p* < 0.001, Cohen’s *d* = 0.63), and control trials and no change trials (FDR *p* < .001, Cohen’s *d* = 0.73). Furthermore, significant performance differences were identified between obvious and subtle different trials (FDR p = 0.005, Cohen’s *d* = 1.41), and obvious different and no change trials (FDR p = 0.004, Cohen’s *d* = 1.44). No other comparisons were significant (Fig 5).

**Fig 6:**
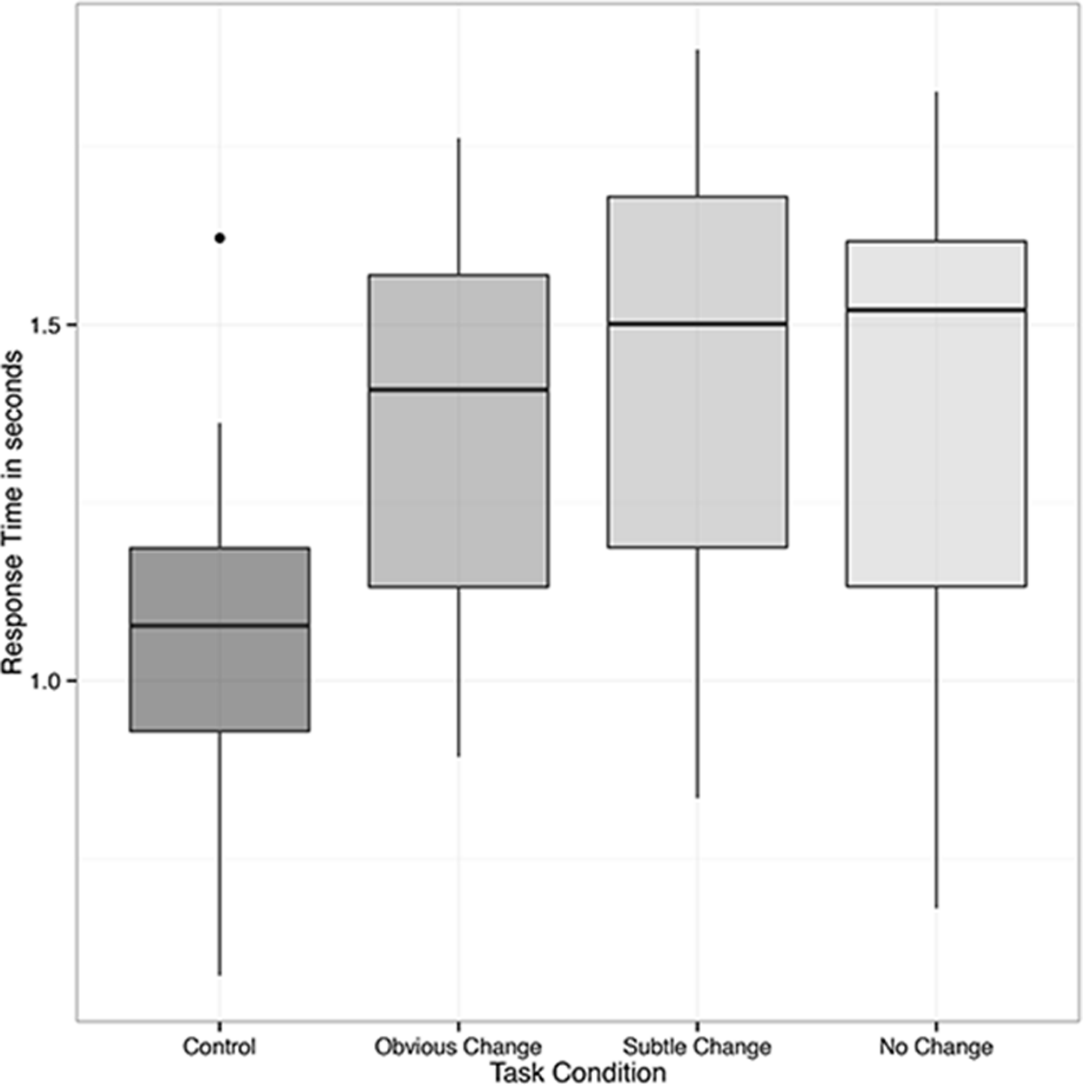
fNIRS task response time. Inter-quartile ranges of fNIRS task response times across each trial type. Similar to the task accuracy outcome, the predicted pattern of faster median trial response times for control trials was identified, followed by “obvious change”, “subtle change”, and “no change” trials in that order.

A similar 2 x 4 repeated measures ANOVA was used to identify task related differences in response time. This analysis identified a main effect of trial type (*F*(3,104) = 7.201, *MSE* = 0.603, *p* = .0002, *partial η*^*2*^ = 0.21). In a pattern similar to the accuracy results reported above, follow-up pairwise t-tests with FDR correction identified significant response time differences between control trials and obvious different trials (FDR *p* = 0.002, Cohen’s *d* = 0.59), control trials and subtle different trials (FDR *p* = 0.0003, Cohen’s *d* = 0.71), and control trials and no change trials (FDR *p* = 0.0008, Cohen’s *d* = 0.67). No other comparisons were significant (Fig 6).

### Cortical activation results

**Fig 7:**
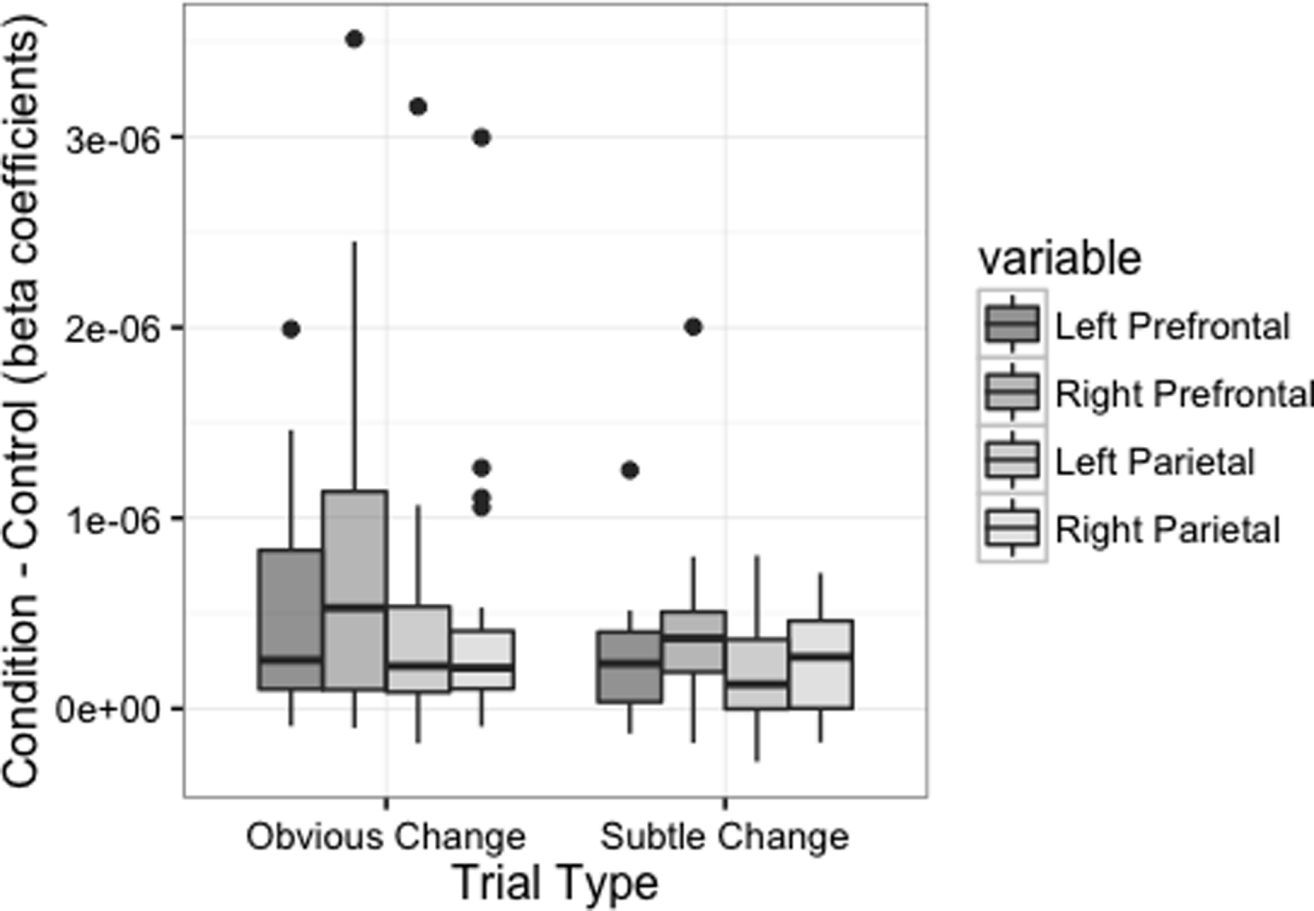
Cortical activation across trial difficulties and regions of interest. Inter-quartile ranges of fNIRS task response times across each trial type. Cortical activation was greater during obvious compared to subtle change trials, and varied significantly across regions of interest.

In order to assess the degree to which cortical activation differed across the trial type (i.e., *subtle different* vs. *obvious different*) and dot number (i.e., 3 vs. 5) task conditions, the beta contrasts calculated for each condition respectively were submitted to a 2 (trial type) x 2 (dot number) x 4 (region of interest) repeated measures ANOVA. This analysis identified a significant main effect of trial type (*F*(1, 208) = 12.197, *MSE* = 3.543^−12^, *p* < 0.001, *partial η*^*2*^ = 0.38), indicating that cortical activation during our task was greater during *obvious* compared to *subtle* change trials. This analysis also identified a significant main effect of region of interest (*F*(3, 208) = 3.742, *MSE* = 1.087^−12^, *p* = 0.012, *partial η*^*2*^ = 0.22), indicating that cortical activation varied across the regions of the cortex we targeted. Follow-up pairwise comparisons indicated that activation in the right PFC was significantly greater than the left (*FDR p* = 0.019) and right (*FDR p* = 0.032) parietal regions, and narrowly missed the rejection criteria for its comparison with the left PFC (*FDR p* = 0.063) (Fig 7).

One aspect of our task that inherently divided the trial type and dot number conditions was the section of each trial at which either condition became apparent to the participant. That is, the dot number condition was apparent to the participant at the beginning of each trial (i.e., during encoding and maintenance), while the trial type condition was not apparent until retrieval. Thus, it is possible that cortical activation in response to 3 and 5 dot trials may differ during the initial portions of each trial (i.e., during encoding and maintenance), and may reduce its effect throughout the latter portions (i.e., retrieval). In order to address this question, we re-estimated beta coefficients for the 3 and 5 dot conditions within the encoding/maintenance and retrieval portions of the task individually, and submitted both to individual 2 (dot number) x 4 (region of interest) repeated measures ANOVAs. For the encoding/maintenance portion of our task, this analysis revealed a significant main effect of dot number (*F*(1, 104) = 4.443, *MSE* = 1.064^−13^, *p* = 0.037, *partial η*^*2*^ = 0.18), indicating that cortical activation was greater for 3 compared to 5 dot trials. The main effect of region of interest was not significant, indicating that each region of interest responded similarly to the 3 and 5 dot conditions. Conversely, an identical analysis conducted on beta coefficients for the 3 and 5 dot conditions estimated for the retrieval condition failed to identify a significant main effect of dot number or region of interest. Taken together, these results suggest that the primary effect elicited by our dot number manipulation occurred within the initial portion of our task trials, but not during retrieval.

**Fig 8:**
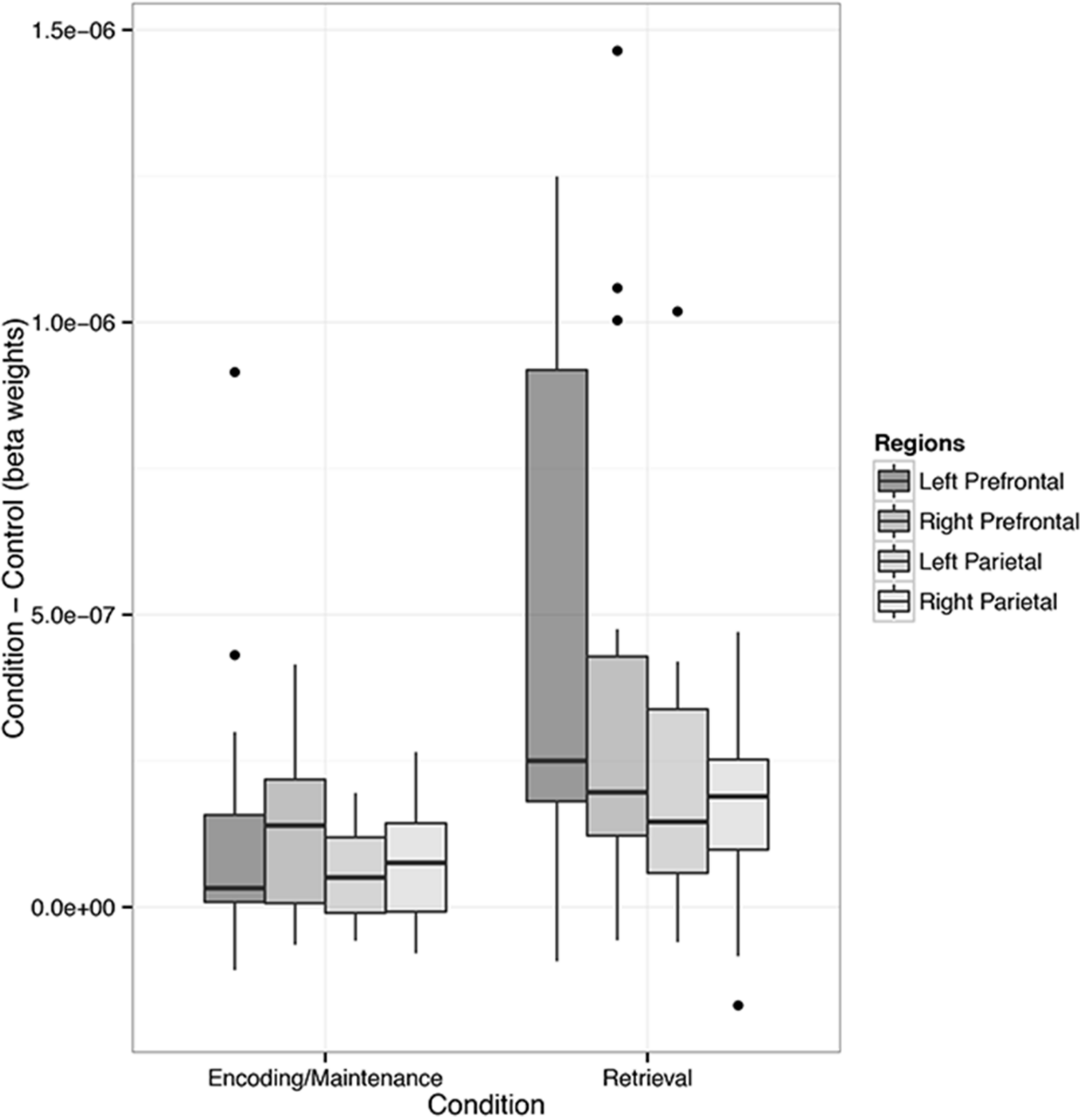
Cortical activation across task conditions and regions of interest. Inter-quartile ranges of cortical activations in response to the encoding/maintenance and retrieval conditions, across each region of interest. Cortical activation was higher during retrieval compared to encoding/maintenance. Moreover, activation was greater in the prefrontal compared to parietal brain regions.

Next, in order to assess the overall patterns of cortical activation that occurred during encoding/maintenance compared to retrieval across each region of interest, we estimated beta coefficients for these sections of all trials (i.e., collapsed across dot number and trial difficulty conditions) prior to conducting a 2 (task: encoding/maintenance vs. retrieval) x 4 (region of interest) repeated measures ANOVA. This analysis identified a significant main effect of task (*F*(1,104) = 14.88, *MSE* = 1.229^−12^, *p* < 0.001, *partial η*^*2*^ = 0.49), indicating that cortical activations across all regions of interest were greater in the retrieval compared to encoding/maintenance trial sections. Furthermore, this analysis indicated that the main effect of brain region approached significance (*F*(3,104) = 2.4, *MSE* = 1.984^−13^, *p* = 0.071, *partial η*^*2*^ = 0.23), but was not robust enough to reject the null hypothesis that activation differed across regions of interest (Fig 8). Finally, FDR corrected one-sample t-tests conducted on the contrast values calculated between the beta weights for non-control trial components compared to control trial components were significant for every contrast in all regions of interest except the left prefrontal region during encoding/maintenance.

### Coherence results

**Fig 9:**
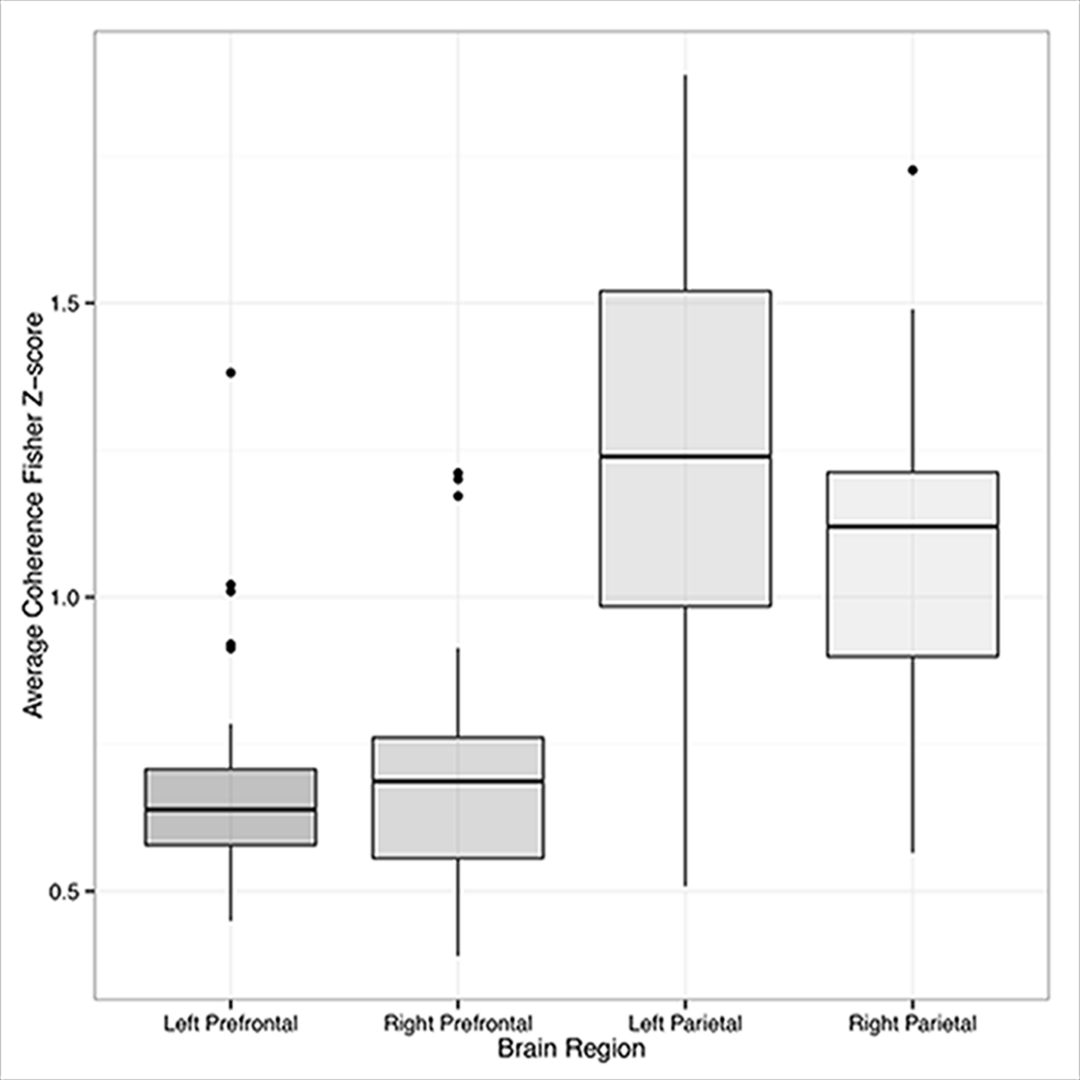
Coherence within regions of interest. Inter-quartile ranges of channel-wise coherence within each region of interest. Because no significant difference in coherence was identified between the encoding/maintenance and retrieval portions of the task, the data shown here have been combined across conditions. Coherence was significantly greater within the parietal compared to prefrontal regions, as well as within same- as opposed to different regions.

**Fig 10:**
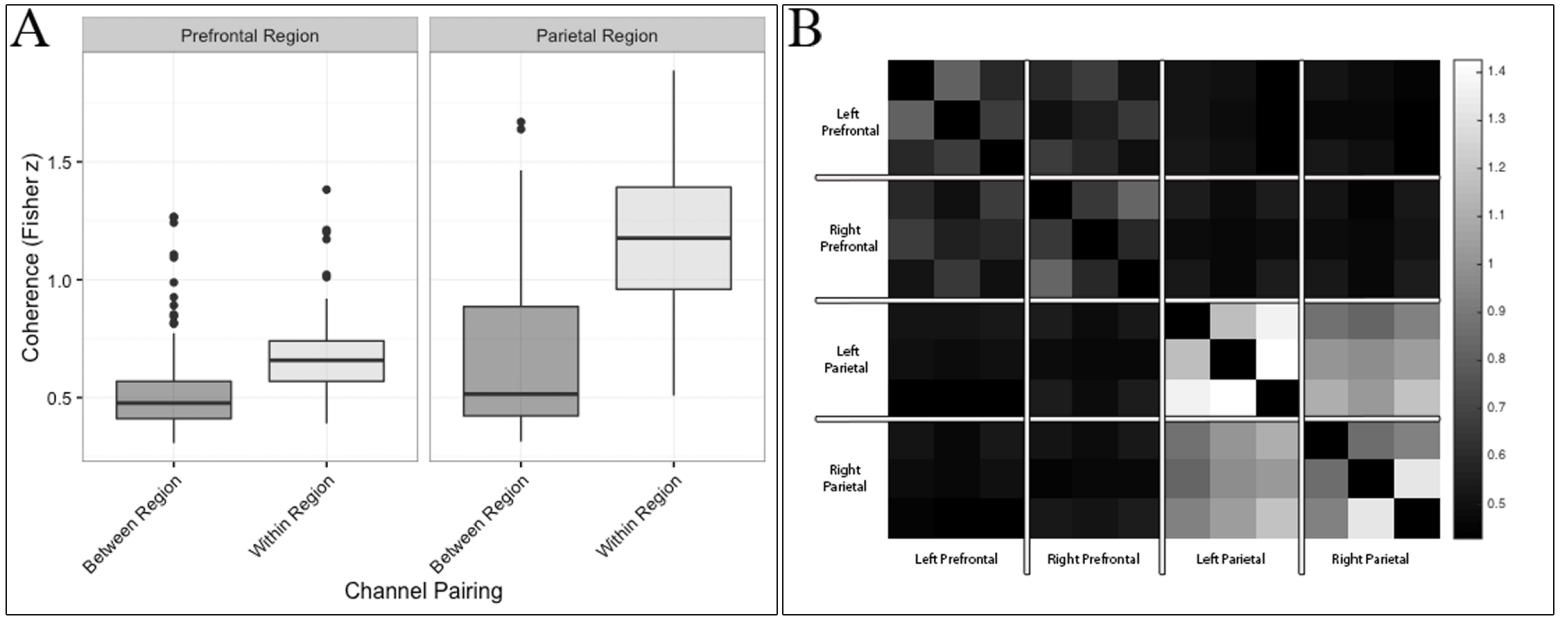
Coherence within and across regions of interest. A. Interquartile range of coherence within and between regions of interest. Coherence was greatest among channels that shared the same region. This effect was greatest among channels within the parietal cortex. B. Heatmap visualization of channel-wise coherence across all regions of interest. The opacity (i.e., whiteness) of each cell indicates the degree of coherence between all (N_channels_ = 12) channels.

In order to assess within- and between-region coherence, respectively, all coherence values were first codified based on their hemisphere (left/right), region (prefrontal/parietal), and coupling (within- or between-region). Next, we conducted a series of one-sample t-tests to determine if the wavelet coherence between all channel pairings was significantly greater than zero during our task. These analyses indicated that task related coherence among channels within each region was greater than zero (FDR *p* < 0.05). Analyses of coherence between regions failed to reject the null hypothesis (FDR *p* > 0.05). To further interrogate this relationship, the coherence values from all channel pairings were submitted to a 2 (side) x 2 (region) x 2 (coupling) x 2 (task: encoding/maintenance vs. retrieval) repeated measures ANOVA. This analysis indicated that task related coherence was significantly greater within the parietal compared to prefrontal regions of interest (*F*(1,464) = 92.460, *MSE* = 6.264, *p* < .001, *partial η*^*2*^ = 0.22) (Fig 9). Moreover, task related coherence was significantly greater in within- compared to between-region channel pairings (*F*(1,464) = 149.982, *MSE* = 10.160, *p* < .001, *partial η*^*2*^ = 0.34). A significant channel region x pairing (within- or between-region coupling) interaction was caused by greater coherence in within-region channel pairs in the parietal region of interest (*F*(1,464) = 35.235, *MSE* = 2.387, *p* < 0.001) (Fig 10). No coherence difference was identified across tasks (FDR *p* > 0.05).

## Discussion

The findings from our study highlight a dissociation between patterns of cortical activation and functional coherence in the frontal-parietal cortices when demands are placed on the visuospatial working memory system. To our knowledge, these are the first data to concurrently identify and assess this dissociation. By combining both approaches, our results help provide a clearer and more complete understanding of the neural correlates to these–and likely other [36]–cognitive demands. Namely, while a given task may elicit significant increases in both cortical activation and functional connectivity, the local maxima of either outcome do not necessarily coincide. Thus, a combined focus on both signatures of brain function together affords a richer and more in depth interpretation of functional brain data.

The findings from our analysis of cortical activation support the findings from previous studies investigating the neural signatures of visuospatial working memory. For example, participants in our task exhibited significant cortical activation throughout the bilateral dorsolateral and superior parietal cortices during our task. These findings support previous research, which identified similar patterns of cortical activity during visuospatial working memory tasks using fMRI [1–3,25,37–40]. Moreover, similar to previous accounts [2], our results indicate that manipulation of dot number significantly influenced cortical activation. Interestingly, in our task this manipulation resulted in greater activation during the three compared to five dot trials. This pattern differs from that reported by Tsujimoto and colleagues (2004), who employed a task similar to ours yet found greater peak oxygenation values in a block averaged time series for five compared to three dot trials. It is possible that our JND estimation procedure induced an encoding/maintenance strategy that differed between dot conditions. That is, while both strategies elicited significant cortical activation, the strategy employed for the three dot condition may have recruited greater neural resources in preparation for the retrieval condition. Alternatively, the five dot condition combined with a possibly difficult perceptual judgment during retrieval may have influenced participant’s attentional resources or stress responses such that cortical activation during this task dropped relative to the three dot condition.

Similar decreases in cortical activation in response to increased cognitive load have been noted previously. For example, increased cognitive load is known to influence selective attention to visual stimuli, resulting in decreased prefrontal activity in high compared to low load conditions [41]. Furthermore, experimentally induced stress has been shown to reduce working memory-related activity in the dorsolateral prefrontal cortex [42]. Thus, it is possible that our JND estimation procedure influenced cortical activation in response to changes in cognitive load differently than classic “fixed difficulty” tasks. Indeed, the same explanation may exist for our seemingly counterintuitive outcomes regarding cortical activation during retrieval. That is, in our task cortical activation was higher during obvious compared to subtle change trials. It is possible that our use of participant-subjective JND to calibrate the difficulty of these trial types influenced participant’s selective attention or induced stress to a greater degree in subtle change trials, causing a decrease in cortical activation relative to the obvious change trials.

On our task, retrieval of visuospatial information elicited greater activation than its encoding and maintenance. With regard to parietal activation, these results are aligned with previous findings, which highlight more prevalent and widespread signatures of activation in the bilateral superior parietal cortices during retrieval compared to encoding [6]. While Spaniol and colleagues were investigating episodic memories, the activation patterns of episodic and working memory are known to have considerable overlap [5]. Furthermore, our results identified a significant main effect of brain region indicating that, on the whole, cortical activations were greater in the prefrontal compared to the parietal cortices. However, closer investigation of activation within each region indicated that the left PFC remained inactive (relative to control trials) during encoding and maintenance of visuospatial stimuli. These results differ from previous reports, which identified significant bilateral activation of the PFC during encoding, maintenance, and retrieval of visuospatial information [6,43]. For instance, d’Espisito and colleagues (1998) reported that verbal working memory demands elicit activation in the left dorsolateral PFC, whereas spatial working memory demands result in activation within the right dorsolateral PFC. As our task relied solely on spatial working memory, our significant results in the right PFC only may be evidence of hemispheric organization of working memory processes in the prefrontal cortex.

Analysis of task-related coherence identified significant within-region coherence for all regions of interest. That is, each channel within a region correlated highly with each of its neighbors within the same region in response to our task. In particular, coherence was higher among channels within the parietal compared to prefrontal cortices, and was greatest in the left parietal cortex. We did not identify significant between-region coherence on our task. Furthermore, in our sample, functional coherence was not influenced by task load (i.e., dot number). This may be due to constraints in our task demands that inhibited load-related changes in functional coherence. Moreover, as our analysis of functional coherence did not include a contrast to a non-task condition, it remains difficult to determine the degree to which our task elicited coherence. An outstanding empirical question is the degree to which task demands from other visuospatial working memory tasks, relative to both rest and changes in task load, influence patterns of functional coherence. Thus, while our results are the first to identify regional and hemispheric asymmetry of functional connectivity in the fronto-parietal neural network during a visuospatial working memory task, future applications that employ different task load demands is warranted.

Notably, a similar pattern of increased coherence within spatially proximate fNIRS channels has also been identified within tests of resting-state connectivity [34,44]. Using a seed-based approach to calculate functional connectivity, Medvedev (2014) identified greater connectivity among channels within compared to between each anatomical region of interest (inferior frontal gyrus and middle frontal gyrus). Consistent patterns of greater coherence within neighboring compared to distant fNIRS channels is likely driven by cerebral blood flow autoregulatory processes that are similar within neighboring brain regions, but may vary significantly across large regions of the brain [45].

An intriguing possibility is that coherence among brain regions fluctuates across tasks and testing environments. For example, recent findings indicate that patterns of functional connectivity differ between resting state and active tasks, as well as between healthy and clinical populations [46–49]. By observing functional connectivity during periods of rest, as well as during the Trail Making Test, Rosenbaum and colleagues (2016) identified significant differences in connectivity throughout the fronto-parietal network between healthy and depressed elderly participants. Interestingly, connectivity strength increased in non-depressed participants, and decreased in depressed participants, as the mental effort required by their tasks increased (e.g., resting mental effort < Trail Making Test mental effort). Thus, functional connectivity may provide a unique indicator of mental effort that is sensitive to changes caused by clinical disorders. Because our current understanding of neural coherence results primarily from highly controlled lab-based studies, it is important that future fNIRS research assess coherence across diverse testing environments that are not accessible by other neuroimaging techniques. Such studies have the potential to elucidate important information about functional processing of real-world stimuli and events in naturalistic settings.

### Future directions and limitations

In this study, we employed multiple methodological techniques that we feel improve the overall validity of our results. First, our JND estimation procedure was used to standardize task difficulty across participants. As discussed above, this procedure likely contributed to our behavioral outcomes. Specifically, behavioral performance on our task was lowest on trials in which no change in the target-to-original location was made. This pattern is due to perceptual ‘false-positives’, which likely occurred because of our task design. That is, the use of subtle different trials near the participants JND may have facilitated a liberally biased response strategy, wherein committing a false positive (i.e., responding that a change occurred when one did not; Type I error) was preferable to committing a false negative (i.e., failing to identify a change that did occur; Type II error). As a result, when presented with a situation in which they were unsure if a change was made, participants opted to respond in the affirmative. However, our task accuracy analysis identified significantly greater performance on obvious different compared to subtle different trials. These outcomes indicate that our JND estimation procedure effectively distinguished difficult and easy trials for all participants regardless of their individual ability level. Taken together with the considerable overlap in cortical activation outcomes between our task and previous studies that employed traditional “fixed difficulty” task designs [1–3,25,37–40], our results provide important justification for the use of pre-scan task calibration measures that provide equal perceived task difficulty for all participants. This is especially important with respect to future examinations of clinical or atypical populations, in which large fluctuations in ability level may exist between participants. It is important that future research be conducted to investigate this issue further.

We introduced a novel method of functional localization that may be used to objectively subset the large numbers of channels typically used in fNIRS studies down to a single channel of interest that can then be entered into group-level statistical analyses. This method differs from common strategies that simply average the data from multiple fNIRS channels. While the use of the International 10/20 is effective in guiding the placement of fNIRS optodes onto anatomical regions of the brain that are likely to be activated by a given task, as well as standardizing such placement across participants, the exact location of the brain within a region of interest that is activated by a task will vary across participants. Thus, a broadly defined region of interest that is covered by multiple fNIRS channels likely contains thousands of neurons that will not activate during a given task, and which vary spatially across participants. Inclusion of these brain regions in the averaging process will mask true patterns of cortical activation elicited by the task. Therefore, our functional localization procedure helps reduce the probability of committing Type II (i.e., false negative) errors. Future research should explore best practices that build from our localization procedure. For example, grouping channels that share a common fNIRS source or detector will inherently constrain the physical distance of each channel within a localization bundle to a spherical region of the cortex with a diameter equal to the fNIRS channel length (e.g., 3cm). Thus, localization of the single highest responding channel with a single “source bundle” (i.e., *source localization*) may be an optimal approach for future fNIRS studies.

Finally, it is important to note that, due to our experimental design, it is difficult to distinguish how the individual components of our task contributed to the pattern of activity that we report during retrieval. This is due to the fixed and short time-interval that separated the encoding/maintenance and retrieval portions of our task. In short, the hemodynamic overlap that occurs between events separated closely in time may mask a true task-related hemodynamic response, such that it will be missed by the GLM procedure [27]. However, because our results show significantly greater activation during retrieval compared to encoding/maintenance, it is likely that any hemodynamic overlap induced by our design was overcome and built upon during retrieval. Moreover, our results demonstrating significant differences between obvious different and subtle different trials, which was not known to the participants prior to the retrieval condition onset, also highlights unique contributions of the retrieval compared to the encoding/maintenance condition. Nevertheless, other cognitive processes that may operate in temporal proximity to a known time point of retrieval (e.g., anticipatory attention, etc.) may influence cortical activity during retrieval. While this issue is commonly overcome by employing a jittered inter-trial interval, our method did not accommodate this design feature. While we do not feel that this significantly affects our results, it is important that future research be conducted to address this shortcoming directly.

## Conclusions

Far from trivial, a clear understanding of the relationship between cortical activation and functional coherence across multiple cognitive tasks has the potential to revolutionize our current understanding of how the human brain accomplishes the myriad demands it faces daily. Here, we show that both signatures are present in the fronto-parietal network during visuospatial working memory, although the location demonstrating the greatest output of either signature do not overlap. Together, these data replicate and extend many existing findings, while providing much needed evidence to help clarify and advance our current understanding of brain function during this ubiquitous process. Combined with the methodological benefits of fNIRS (e.g., portability, tolerance to movement, etc.), future studies may employ our methods to interrogate these concurrent signatures in more naturalistic environments.

## Supporting information

S1 File Deoxygenated data analysis. Identical cortical activation analyses as reported in the main text for deoxygenated fNIRS data.
(DOCX)

S2 File Behavioral data. Task behavioral data. The number of dots in a trial is given in the “numDots” column; Trial response time is given in the “RT” column; Trial type is given in the “TrialType” column; Trial accuracy is given in the “TrialAcc” column.
(CSV)

S3 File Cortical activation data. fNIRS data used for cortical activation analyses. Subject-level beta contrasts are given for each participant region of interest, and data type (HbO and HbR). The contrast of interests is given in the “Contrast” column; The data used is given in the “Data” column; Regions of interest (left/right PFC & Parietal regions) are distributed across columns.
(CSV)

S4 File Coherence data. fNIRS data used for functional coherence analyses. Subject-level mean coherence values are given for each task and region of interest.
(CSV)
