# Correction: fNIRS measurement of cortical activation and functional connectivity during a visuospatial working memory task

**DOI:** 10.1371/journal.pone.0203233

**Published:** 2018-08-24

**Authors:** Joseph M. Baker, Jennifer L. Bruno, Andrew Gundran, S. M. Hadi Hosseini, Allan L. Reiss

The image for Fig 1 is incorrect. Please view the correct Fig 1 here.

**Fig 1:**
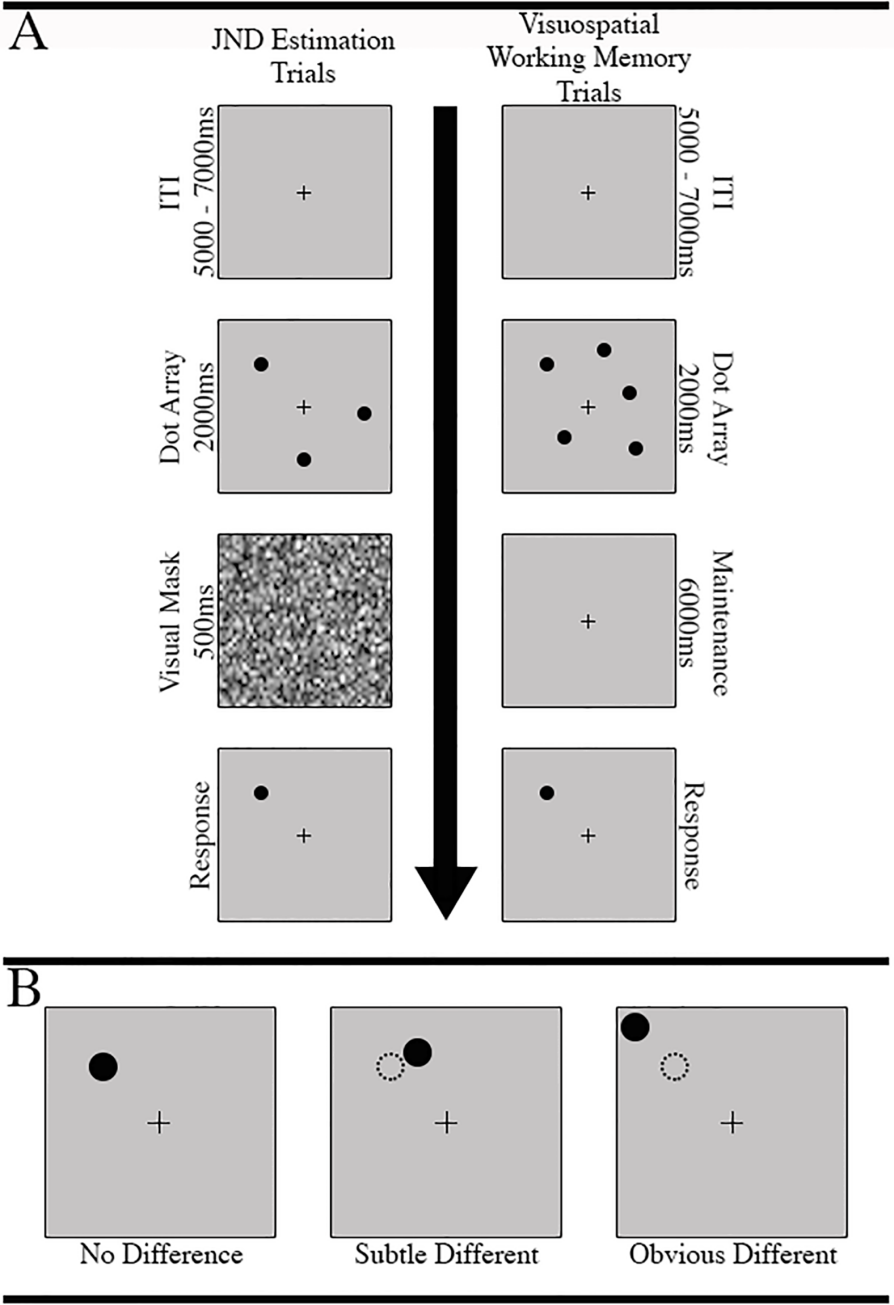
Trial structure for JND and fNIRS task trials. A. Participants completed 200 JND estimation trials. Each trial began with a 5–7 second ITI, and was followed by a 3 or 5 dot array. Next, a visual mask was displayed for .5s, and was immediately followed by the single-dot presentation, at which point the participant could respond. The fNIRS task trails proceeded in a similar fashion, except the visual mask portion was extended to 6s and the screen simply remained blank except for a central cross; B. fNIRS task difficulty was modulated by the spatial difference between the original target dot location within its array and its location on the response screen. Spatial deviations were defined as “no difference”, “subtle difference” (JND x 0.1), and “obvious difference” (JND x 0.8). The updated location of the target dot was randomly selected from a vector of x- and y-axis values that constitute a circle, with a radius equal to the spatial deviation in pixels, that lay around the center of the original dot.
