# Supplementary material for: fNIRS measurement of cortical activation and functional connectivity during a visuospatial working memory task
Source: PLoS One. 2018 Aug 2;13(8):e0201486. doi: 10.1371/journal.pone.0201486 (PMC6072025; doi:10.1371/journal.pone.0201486)
Supplement: S1 File — Identical cortical activation analyses as reported in the main text for deoxygenated fNIRS data. (DOCX) [file pone.0201486.s001.docx]

fNIRS measurement of cortical activation and functional connectivity

during a visuospatial working memory task

______________________________________________________________________________

SUPPORTING INFORMATION: DEOXYGENATED DATA ANALYSIS

Identical cortical activation analyses were conducted for the HbR data. All preprocessing and GLM parameters reported in the manuscript were kept constant for the HbR analyses. A 2 (trial type) x 2 (dot number) x 4 (region of interest) repeated measures ANOVA identified a significant main effect of region of interest (F(3, 208) = 3.678, MSE = 1.293e-13, p = 0.013). These results coincide with an identical main effect in the HbO analysis. However, unlike the HbO analysis, no other comparisons were significant within the HbR data. Taken together, these results indicate that both sources of fNIRS data provide overlapping information about the cortical response to our task, although for our task HbR is less robust compared to HbO.
